# Induction of *Fusarium* lytic Enzymes by Extracts from Resistant and Susceptible Cultivars of Pea (*Pisum sativum* L.)

**DOI:** 10.3390/pathogens9110976

**Published:** 2020-11-23

**Authors:** Lakshmipriya Perincherry, Chaima Ajmi, Souheib Oueslati, Agnieszka Waśkiewicz, Łukasz Stępień

**Affiliations:** 1 Plant-Pathogen Interaction Team, Department of Pathogen Genetics and Plant Resistance, Institute of Plant Genetics, Polish Academy of Sciences, 60-479 Poznań, Poland; 2 Biological Engineering/Polytechnic, Université Libre de Tunis (ULT), Tunis 1002, Tunisia; chaima.ajma@outlook.com; 3 Laboratoire Matériaux, Molécules et applications, Institut Préparatoire aux Etudes Scientifiques et Techniques, La Marsa 2070, Tunisia; souheibo@yahoo.fr; 4 Department of Chemistry, Poznań University of Life Sciences, 60-625 Poznań, Poland; agnieszka.waskiewicz@up.poznan.pl

**Keywords:** CWDEs, fumonisins, *Fusarium*, lytic enzymes, plant–pathogen interaction

## Abstract

Being pathogenic fungi, *Fusarium* produce various extracellular cell wall-degrading enzymes (CWDEs) that degrade the polysaccharides in the plant cell wall. They also produce mycotoxins that contaminate grains, thereby posing a serious threat to animals and human beings. Exposure to mycotoxins occurs through ingestion of contaminated grains, inhalation and through skin absorption, thereby causing mycotoxicoses. The toxins weaken the host plant, allowing the pathogen to invade successfully, with the efficiency varying from strain to strain and depending on the plant infected. *Fusarium* *oxysporum* predominantly produces moniliformin and cyclodepsipeptides, whereas *F*. *proliferatum* produces fumonisins. The aim of the study was to understand the role of various substrates and pea plant extracts in inducing the production of CWDEs and mycotoxins. Additionally, to monitor the differences in their levels when susceptible and resistant pea plant extracts were supplemented. The cultures of *F. proliferatum* and *F. oxysporum* strains were supplemented with various potential inducers of CWDEs. During the initial days after the addition of substrates, the fungus cocultivated with pea extracts and other carbon substrates showed increased activities of *β*-glucosidase, xylanase, exo-1,4-glucanase and lipase. The highest inhibition of mycelium growth (57%) was found in the cultures of *F. proliferatum* strain PEA1 upon the addition of cv. Sokolik extract. The lowest fumonisin content was exhibited by the cultures with the pea extracts and oat bran added, and this can be related to the secondary metabolites and antioxidants present in these substrates.

## 1. Introduction

Pea (*Pisum sativum* L.) is a major legume crop, cultivated mainly for its high quality vegetable protein and rich essential amino acid content [1]. The amino acid content of pea seed is characterized by the high amount of leucine, isoleucine and arginine, but low of methionine, cysteine and tryptophan [2]. Therefore, it is estimated that this crop can substitute high protein animal meat products. Additionally, the pea plants play an important role in *Rhizobium*-mediated nitrogen fixation in the soil [3]. Pea plant is susceptible to various soil-borne fungal diseases such as foot, root and collar rots caused by *Fusarium oxysporum, Rhizoctonia solani, Sclerotinia sclerotiorum, Thielaviopsis basicola* and *Pythium* spp. [4]. The species from the *Fusarium* genus: *F. oxysporum* f.sp. *pisi, F. oxysporum* var. redolens, *F. poae, F. solani* and *F. avenaceum* are considered as minor pathogens of pea [5,6], nevertheless they can often cause the accumulation of mycotoxins in plant tissues [7] and are the main causes of crop failures. Often, short crop rotations result in severe disease in the fields. Disease symptoms include chlorotic lesions, leaf curling and turning flaccid, which eventually wilts. The initiation of the root infection occurs when the fungus penetrates the roots and moves towards the vascular tissues [8]. Successful penetration involves the action of the complex of various cell wall-degrading enzymes (CWDEs), which are a combination of lignocellulases [9], proteases [10] and lipases [11]. The fungus moves to the neighboring tissues by means of mycelia, conidia or by microconidia, and extensively invades the upper parts of the susceptible plant [8]. In contrast, the infection is limited to the basal part of the resistant varieties [12]. Based on a previous study, the pea extracts were found to inhibit the biosynthesis of fumonisins B in *F. proliferatum* strains and also limit the fungal biomass production, which is attributed to the action of various plant secondary metabolites, including pisatin—a phytoalexin produced in pea as a defense response to *F. oxysporum* [13,14]. Hence, we selected susceptible and resistant cultivars of pea, namely Santana and Sokolik, and their extracts were used for the lytic enzymes’ induction.

CWDEs are produced by the plant–pathogenic fungi and enable them to penetrate and infect the host tissue [11,15,16,17,18]. They contribute to the degradation of waxy cuticle and cell walls. The pathogen has to force the cuticular waxes covering the host epidermal cell during the first encounter [15]. The key cell wall lytic enzymes of pathogenic fungi are chitinases and *β*-1,3-glucanases. *F. oxysporum* depolymerize all components of plant cell walls, such as cellulose, xylan, pectin, polygalacturonic acids and proteins (extensins) [19]. Along with the production of enzymes, the fungi synthesize mycotoxins in order to weaken the host defense machinery. These mycotoxins reach the animals through contaminated feed and fodder and also human beings through contaminated grains, other food products, dermal exposure or through inhalation. An example is fumonisin B_1_ produced by *F. proliferatum* that causes liver and esophageal cancer and pulmonary weakness in humans and animals [20]. They disrupt plasma membranes of both plants and animals by the accumulation of toxic sphingolipid intermediates. De-novo sphingolipid biosynthesis is interfered by inhibiting the enzyme ceramide synthase by these intermediates, thus disrupting cell signaling, cellular apoptosis and replication [21,22]. Toxins, such as beauvericins and enniatins, induce DNA fragmentation and apoptosis, by disrupting the mitochondrial pathways [23]. Furthermore, beauvericins reduce the level of ascorbic acid in the cell, thereby collapsing the ascorbate system and leading to protoplast death in tomatoes [24]. Moniliformin is another toxin that is less toxic than trichothecenes and is produced by *F. proliferatum, F. oxysporum, F. avenaceum, F. subglutinans, F. chlamydosporum* and *F. anthophilum* [25]. The main objectives of the study were to determine comparatively the effects of various carbon sources and extracts obtained from susceptible and resistant pea cultivars on the production of extracellular cell wall-degrading enzymes by *F. proliferatum* and *F. oxysporum* strains, and to determine their effects on mycotoxin production in liquid in vitro cultures. The study also helps to understand how the resistant and susceptible pea cultivars differ in altering the fungal biology. The action of CWDEs and mycotoxins during the plant–pathogen interaction is a better way of concluding their possible roles in plant pathogenesis.

## 2. Results

### 2.1. Dry Mycelial Biomass

**Figure 1:**
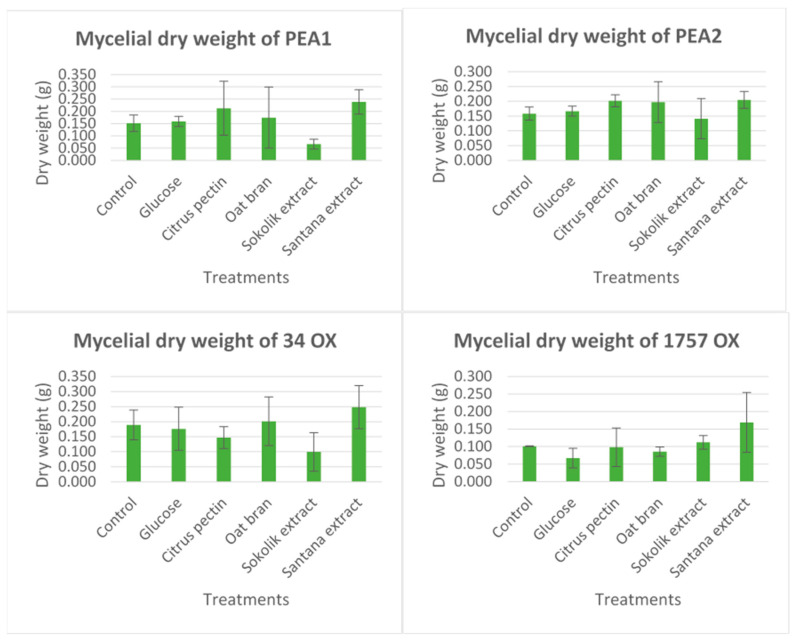
Dry mycelial biomass (in grams) of *F. proliferatum* (PEA1 and PEA2) and *F. oxysporum* (34OX and 1757OX) strains on 14th day of culturing with various substrates and pea extracts (error bars represent standard error).

Among all the substrates used, Sokolik extract was the most effective in inhibiting the fungal growth, whereas Santana showed the exactly opposite activity. All the comparisons were done based on an absolute control. For instance, the mean weight of mycelial dry mass of PEA1 and PEA2 strains with the extracts of Santana was 239 mg and 205 mg, respectively (Figure 1). The corresponding dry mass of the Sokolik extract-added cultures were 66 mg and 141 mg. All other substrates showed no significant inhibition on the mycelial biomass. A similar result was obtained in 34OX too, but a small increase in mean mycelial mass has been observed in the case of Sokolik extract-added cultures of 1757OX compared to the control. However, the difference observed was statistically insignificant (*p* > 0.05).

### 2.2. Induction of Extracellular Enzymes

**Figure 2:**
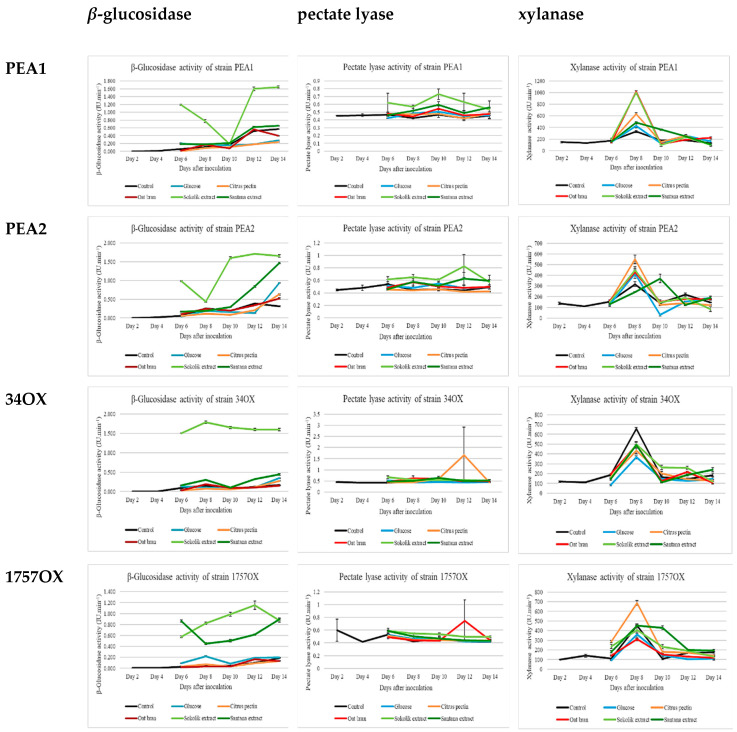
*β*-glucosidase (micrograms of P-nitrophenol produced per minute (U/min)), pectate lyase (micrograms of galacturonic acid produced per minute (U/min)) and xylanase (micrograms of xylose produced per minute (U/min)) activities of PEA1, PEA2, 34OX and 1757OX strains cultures from day 1 to day 14 of culturing. Calculated from triplicate treatments. Error bars represent standard error.

The activity assays of various cell wall-degrading enzymes, such as xylanase, protease, lipase, polygalacturonase, pectin lyase, chitinase, endo β-1,4 glucanase (CMCase), exo-β-1,4-glucanase (avicelase), β-glucosidase and cellulase (filter paper assay) were studied. The extracts from two pea cultivars (Sokolik and Santana) induced increased activity of several enzymes, prominently β-glucosidase, pectate lyase and xylanase (Figure 2). Data from the statistical analysis of β-glucosidase, pectate lyase and xylanase activities on the 8th day of all fungal strains supplemented with Sokolik and Santana extracts are given in Table 1. The tables and graphical representations of protease, lipase, polygalacturonase, chitinase, endo β-1,4 glucanase, exo-β-1,4-glucanase and cellulase enzyme activity results are presented as Supplementary Materials (Tables S1–S7 and Figures S1–S7).

#### 2.2.1. Induction of Extracellular Enzymes in F. proliferatum Cultures 

The statistical data indicates that Sokolik-added cultures show significant increase in the activity of enzymes such as β-glucosidase, pectate lyase and xylanase in PEA1 and PEA2 cultures on 8th day (*p* < 0.05). The extracellular cell wall-degrading enzymes were induced, which suggests that there is a gradual increase in the overall activity of cellulase enzymes, importantly for β-glucosidase and exo-β-1,4-glucanase in all *F. proliferatum* cultures supplemented with pea extracts. However, the difference observed for exo-β-1,4-glucanase was statistically insignificant (*p >* 0.05) Increased activity of xylanase and pectate lyase was observed in the *F. proliferatum* cultures with pea extracts. Especially, there was a sudden increase in the xylanase activity on day 8 in PEA1 cultures. The Sokolik- and oat bran-supplemented cultures showed more than a 3-fold increase in activity compared to the control, whereas reduced activity was shown for citrus pectin, Santana extract and glucose added to the cultures. A slight increase in the pectate lyase activity was found in cultures supplemented with pea extracts. This activity was maintained throughout the whole culture period. The *β*-glucosidase activity in all PEA1 cultures increased, starting from day 1, especially when the substrates were added on the 5th day. The cultures supplemented with pea extracts showed immediate increase in activity, then there was a sudden fall in the activity on day 10, and again increase on the next day. In contrast to the PEA1 cultures, the activity levels in PEA2 did not drop on the 10th day. Instead, they gradually increased every day. Similarly to PEA1, the Sokolik extract-added PEA2 cultures showed substantial increase in the activity, i.e., twice the activity than any other supplements.

A sudden increase in the lipase activity was observed in all the substrate-added cultures when compared to control in PEA1 on the 6th day, which continued until the 12th day. Here, an exceptional increase in the activity was found in the pea extract-added cultures, which gradually increased and maintained its activity until day 12 and decreased on the final day. In the case of PEA2 strain the highest peak in activity was found in the pea extract-added cultures on the 6th day, which dropped down in the consecutive day and continued this pattern on intermittent days. On day 8, the Sokolik- and Santana-added cultures of PEA1 and PEA2 showed increased activity and the results were statistically significant (*p* < 0.05). No significant protease activity was exhibited by both strains. However, the cultures supplemented with Sokolik extract, which showed an elevation in activity on day 6, reduced on the consecutive days. The polygalacturonase activity of both strains remained insignificant throughout the whole culture period indicating that there was no/very low activity exhibited by the substrates. In PEA2 cultures, both pea extract-added cultures showed a slight increase in the activity. Slow but steady increase in the chitinase activity was exhibited by the PEA1 culture supplemented with the Santana extract, while the cultures with Sokolik, oat bran and citrus pectin showed reduced activity on days 8 and 10 when compared to the control. In the case of PEA2, comparatively reduced activity was obtained in the extract-added cultures, whereas oat bran and glucose-added cultures showed increased activities on day 8 and day 10, respectively.

Cellulases are classified into several enzymes, like endo-1,4-*β*-glucanase, exoglucanase and *β*-glucosidase [18], and are an integral part of the CWDEs. It has been observed in *F. proliferatum* that the activity of the endo *β*-1,4 glucanase in glucose, oat bran and pectin-added cultures showed an elevation during the 8–14 day period, which suggests that there is a comparatively slow reaction of the fungi to the substrates. However, the results were not statistically significant (*p* > 0.05). In the case of exo-*β*-1,4 glucanase, the activity was found to be fluctuating and decreased every intermittent day in PEA1 cultures, but there was a slight increase in the activity in the pea extracts-added cultures. The results for PEA2 were similar, the Sokolik-added cultures showed a two-fold increase in the activity on day 8. However, the activity improved slowly on the 12th and 14th days in the Santana-added cultures. It was worth noting that the cellulase enzyme (FPase) activity of the PEA1 dropped down to the control levels immediately after the addition of substrates, and they continued until the day 14, with a slight increase in the day 10 for the culture supplemented with oat bran. A statistically significant reduction in the activity (*p* < 0.05) was observed for pectin, Sokolik- and Santana-added cultures of PEA1 on day 8. In contrast, on day 12 the cultures of PEA2 with Santana extract, pectin and oat bran, showed 2-fold, 2.5-fold and 3-fold increase in activity when compared to the control, respectively, and again dropped to the level of control on day 14.

#### 2.2.2. Induction of Extracellular Enzymes in F. oxysporum Cultures

There was a gradual increase in the activity of enzymes that can be attributed to the difference in the strains and also to the type of the substrate used as the enzyme inducer. Similarly to the *F. proliferatum* cultures, the xylanase activity of *F. oxysporum* cultures also got a peak on the 8th day (statistically significant (*p* < 0.05)) with an exception of decreased activity in all substrate-added cultures, when compared to the control of the 34OX strain, whereas the activity gradually increased 2-fold for consecutive 4 days in the Sokolik-added cultures. The 1757OX cultures supplemented with citrus pectin showed about 700 U of activity, where others showed less than 450 U of activity on the 8th day. In contrast to the other strain, Santana-added cultures showed about 2-fold elevated activity. A slight increase in the pectate lyase activity was found throughout the whole culture period in the cultures supplemented with the pea extracts. The cultures supplemented with pea extracts showed increased activity on the 8th day when compared to the control and all other substrates and the results were statistically significant (*p <* 0.05). The β-glucosidase activity was 15-fold higher from day 6 to day 14 in Sokolik-added 34OX cultures compared to the control. Similarly, the activity was double the control in Santana-added cultures, too. In contrast, the 1757OX strain had the highest β–glucosidase activity with Sokolik extract on day 12, and with Santana extract on days 6 and 14. The reaction to Sokolik extract was found to be immediate and reduced gradually over time. On the other hand, the reaction to Santana extract was somewhat slower when compared to Sokolik.

The lipase activity expressed by both *F. oxysporum* strains peaked upon the addition of pea extracts, especially when Sokolik extract was added. Especially, the strain 1757OX supplemented with Sokolik extract showed significant increase in the activity on 8th day when compared to all other substrates (*p* < 0.05). The polygalacturonase activity of 34OX overlapped with the control on intermittent days until 12th day, when the pectin- and glucose-added cultures showed increased activity. In the cultures of 1757OX with Sokolik extract, oat bran, pectin and glucose, distinct activity during 8th, 10th and 14th days were exhibited, and were also statistically valid (*p* < 0.05). No significant protease activity (*p* > 0.05) was exhibited by both strains, except for the cultures supplemented with Sokolik and Santana extracts, showing an elevation on day 6, which was reduced on the consecutive days, similarly to *F. proliferatum*. The activity in both *F. oxysporum* cultures supplemented with Sokolik peaked for the first two days and decreased gradually. Similarly to PEA2, in 34OX there was comparatively reduced chitinase activity in both pea extract-added cultures throughout the culture period, especially, on day 10, where the activity was reduced drastically compared to the control. In contrast to the 34OX, there was a slight increase in the activity on day 10, 12 and 14.

The activity of endo-*β*-1,4 glucanase in the cultures of the 34OX was increasing every day irrespectively of the substrates added, whereas continuous variation in the activity was observed in the case of Santana extract-added 1757OX cultures. However, the results were statistically insignificant (*p* > 0.05). No/extremely low activity of exo-*β*-1,4 glucanase was observed in 34OX cultures. Nevertheless, all the cultures, except for the glucose-added ones, showed increased exo-*β*-1,4 glucanase activity during the first few days in 1757OX. The Santana-added cultures showed two-fold increase in activity throughout the culture period when compared to the control. In the case of total cellulase activity (FPase), both *F. oxysporum* strains showed similarities to the PEA1 strain with less activity for the pea extract-added cultures, and increased activity in glucose/pectin-added cultures.

### 2.3. Fumonisin Quantification

**Figure 3:**
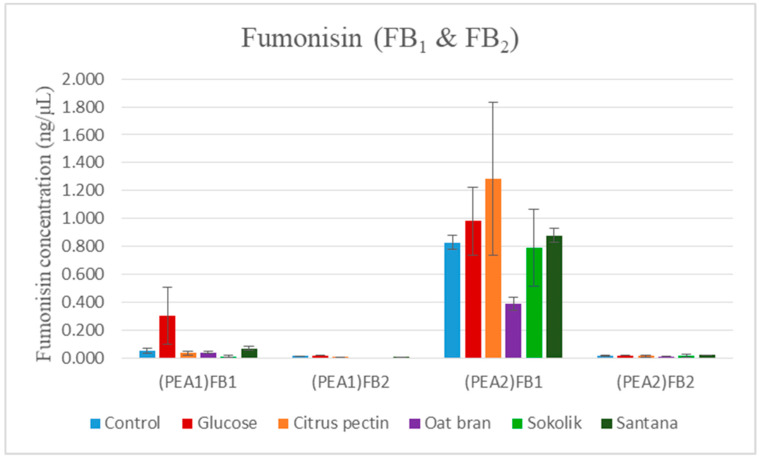
FB_1_ and FB_2_ produced (ng/µL) in PEA1 and PEA2 liquid cultures upon addition of glucose, citrus pectin, oat bran, Sokolik extract and Santana extract. Calculated from triplicate treatments.

The mycotoxins produced by PEA1, PEA2, 34OX and 1757OX in rice cultures are given in Table 2. In the liquid cultures fumonisins B_1_ and B_2_ were produced by both strains of *F. proliferatum* and their synthesis highly depended on the type of substrate used (Figure 3). The amounts of fumonisin B_3_ were always below detection limits (data not shown). Very low traces of FB_1_ and FB_2_ were detected in *F. oxysporum* cultures, too (Table 3). Production of FB_1_ and FB_2_ in both *F. proliferatum* strains was comparatively lower in cultures supplemented with oat bran, citrus pectin, Sokolik and Santana extracts. No FB_2_ was detected in PEA1 cultures supplemented with oat bran and Sokolik extract, very low production was observed in pectin, glucose and Santana-added cultures of PEA1. Extremely low quantities of FB_2_ were detected in both PEA1 and PEA2 cultures supplemented with citrus pectin, oat bran, glucose, Sokolik and Santana extracts. Similar results were obtained for FB_1_ quantification of PEA1. In contrast to all three sets, higher quantities of FB_1_ were produced in PEA2 cultures irrespective to the substrate. Approximately 5.6× reduction was obtained in Sokolik-added cultures compared to the control. Oat bran and citrus pectin could also reduce the fumonisin content for about 2 times. The statistical analysis was carried out using the SAS software assuming *p* value < 0.05 and the results showed that the differences were not statistically significant (amount of FB_1_ and FB_2_ produced in *F. proliferatum* cultures (mean and standard error) are given in Supplementary Materials Table S8).

## 3. Discussion

Biological control of the soil-borne pathogen offers a better and cost-effective alternative to chemicals and is environmentally safe [26]. It is also important to know their mode of action to understand their potential. Here, various stress inducers were used to determine their effects on the induction of various lytic enzymes in the fungus. It is noteworthy that the pea extracts could effectively reduce the mycelial mass of the *Fusarium* species. Similar results were reported earlier, which indicates that the extracts could inhibit pathogenic fungal growth [27,28]. Some reports suggest that pea chitinases and *β*-1,3-glucanase strongly inhibit growth of a wide range of potentially pathogenic fungi [28]. The exudates of several resistant cultivars of pepper and wild chickpea were also found to have inhibition properties, which were related to the production of undetermined phenolic compounds and amino acids, such as methionine, d-1-*β*-phenylalanine, citrulline and d-xylose [29]. Cell wall-degrading enzymes are produced by pathogenic fungi in order to degrade the complex cell wall and to establish the pathogen inside the plant cells. The lytic enzymes also have great importance in commercial production of biofuels and other bioproducts. In the commercial aspect, the recalcitrance of the plant lignocellulosic components is a huge hindrance and the technologies are very expensive due to the high cost of hydrolytic enzymes [30]. However, studies using pathogenic and non-pathogenic fungi on various carbon sources have proven that the pathogenic fungal species, such as *Fusarium* and *Phytophthora,* had higher xylanase activity than many other non-pathogenic fungi, such as *Trichoderma, Penicillium* and *Chaetomium* [31]. Similarly, increased production of xylanase was obtained in cultures supplemented with citrus pectin as the sole carbon source. In our study, the activity was found to be higher on the 8th day of culture, which denotes that the fungi prominently produce xylanases during mid-exponential phase, which was already reported before [32]. Enzymes such as *β*-glucosidase, xylanase, exo-1,4-glucanase and lipase were the first ones to have increased activity after the addition of pea extracts and other carbon substrates. All four strains showed very high activity of *β*-glucosidase once the pea extracts were added, indicating that *β*-glucosidase is one of the most synthesized enzyme for successful penetration in the host plant by these species. This activity was found to be one of the characteristics of *F. graminearum, F. phaseoli, F. proliferatum* and *F. solani,* except for *F. oxysporum*, which exhibited moderate activity as per the report of [33]. All the *F. oxysporum* cultures supplemented with carbon sources, such as glucose, pectin and oat bran, showed reduced/moderate activity similar to the control. In contrast, about 20× increased activity was observed in the Sokolik extract-added cultures. It was quite intriguing to notice that the polygalacturonase activity of *F. proliferatum* cultures supplemented with citrus pectin was slightly lower when compared to the control, whereas a slight increase in activity was observed in the cultures supplemented with the pea extracts. It was reported earlier that, although production of pectinolytic enzymes is not essential for the development of wilt symptoms, it might still play some roles in pathogenicity of the fungus [34]. All four strains showed reduced activity of both cellulase and chitinase, even after the addition of substrates and extracts. Exceptional increase of *β*-glucosidase, xylanase and pectate lyase activities with 4–6-fold differences observed in the cultures supplemented with Sokolik extract, possibly attribute to the higher resistance level of the cultivar to fungal infection. It was understood that metabolites from both cultivars were recognized differently and activated specific signaling pathways, and the genes encoding lytic enzymes necessary for host tissue degradation (authors’ studies, unpublished).

The mycotoxin quantification indicated that both *F. proliferatum* strains were capable of producing fumonisins and their concentration is substrate-dependent. The production of various mycotoxins by *Fusarium* species highly depends on the strain and the host/substrate used for their growth [35]. Even though *F. proliferatum* and *F. oxysporum* were known to produce beauvericins and enniatins [36,37], the mycotoxins were not detected in the liquid cultures in this study. Rather, they were found be synthesized in the rice solid state cultures. Significant variations were obtained between the amounts of FB_1_ and FB_2_ in rice cultures and liquid cultures. According to the previous studies, it was found that host plant extracts could modulate the biosynthesis of fumonisin in *F. proliferatum* [13,14]. Extracts of garlic, pineapple asparagus and pea could alter the *FUM1* gene expression and, thereby, the levels of fumonisins. Similarly, it was found that the pea extract could reduce the level of fumonisins and also inhibit the growth of the fungus [14]. Therefore, the study provides the evidence that the metabolites in the pea have a strong antifungal effect and could effectively reduce the amount of toxins produced. These compounds are not toxic to *Fusarium,* rather they are capable of altering the metabolism. One of the secondary metabolite in pea called pisatin was and its antifungal activity, and the role of pisatin demethylase produced by pathogenic fungi that degrade pisatin was discussed previously [13]. Quite interesting was to find that the oat bran could reduce the fumonisin production similarly to the pea extracts, even though they are susceptible to *Fusarium* infestations [38]. The antioxidants present in oats may have a possible role in reducing the toxin levels in the cultures. They include: tocopherols, sterols, tocotrienols, avenanthramides (unique to oats), p-hydroxybenoic acid, sinapic and vanillic acid. In these, sublethal doses of α-tocopherols could alter the fumonisin biosynthesis [39]. Avenenthramides exclusively present in oats are a type of phytoalexins that could be another reason for reduced fumonisin production. The role of these compounds in regulating mycotoxin biosynthesis has to be further analyzed for better understanding the changes in the pathogen while encountering these metabolites.

## 4. Materials and Methods

### 4.1. Fungal Strains and Growth Conditions

Two strains of *F. proliferatum* (PEA1 and PEA2) and two strains of *F. oxysporum* (34OX and 1757OX) were used for the study. All the strains used for the study were isolated from infected pea plants (*Pisum sativum* L.). The strains were selected out of seven strains preliminary tested, based on their ability to produce mycotoxins in rice cultures. The PDA-grown 7-day-old cultures were inoculated into autoclaved rice and were allowed to grow for 20 days. Later, the whole culture was air dried and ground in to fine powder with a blender, and served as the sample for further mycotoxin analysis. The strains PEA1 and PEA2 produced high quantities of fumonisins B, whereas 34OX and 1757OX strains produced detectable quantities of beauvericin. Fungal strains were cultured on potato dextrose agar (PDA) at 26 °C. Mycelium of about 4 cm^2^ from the 7-day-old culture were added to 250 mL Reyes and Byrde medium and incubated without shaking at room temperature. The medium contained yeast extract 2 g/L, KH_2_PO_4_ 1 g/L, MgSO_4_·7H_2_O 0.5 g/L, KCl 0.5 g/L, (NH_4_)_2_SO_4_ 0.5 g/L and 1 mL of microelement solution (Na_2_BO_7_·10H_2_O 100 mg/L, CuSO_4_·5H_2_O 10 mg/L, FeSO_4_·7H_2_O 50 mg/L, MnSO_4_·5H_2_O 10.8 mg/L, (NH_4_)_6_·Mo_7_O_24_·4H_2_O 10 mg/L and ZnSO_4_·7H_2_O 70 mg/L). Similarly, the strains were inoculated to the fumonisin inducing media for mycotoxin analysis [40]. The medium contained: malt extract 0.5 g/L, yeast extract 1 g/L mycological peptone 1 g/L, KH_2_PO_4_ 1 g/L, MgSO_4_·7H_2_O 0.3 g/L, KCl 0.3 g/L, ZnSO_4_·7H_2_O 0.05 g/L, CuSO_4_·5H_2_O 0.01 g/L and D-fructose 20 g/L. On the 5th day of incubation, 0.05% (*w*/*v*) glucose, citrus pectin and oat bran, and 10 mL each of the extracts from Sokolik and Santana cultivars were supplemented to the cultures. A control was also kept without the addition of any supplements. All the treatments were carried out in triplicates. The culture media were collected in 10 mL aliquots on the 6th, 8th, 10th, 12th and 14th day of incubation and centrifuged to collect the supernatant. They were stored at −80 °C for the enzyme activity analysis and mycotoxin analysis. Total mycelia were collected on the 14th day, frozen in liquid nitrogen and freeze-dried immediately using a lyophilizer until the water is completely removed from the samples. After the complete lyophilization, the weight of each samples were measured.

### 4.2. Preparation of Plant Extracts

Plant extracts were prepared according to the previously standardized protocol [14]. Leaves from fully-grown pea plants (Sokolik and Santana) were frozen overnight at −80 °C and were homogenized using blender. The obtained pulp was centrifuged at 12000× *g* for 15 min. The supernatants were filtered using a 0.45 μm membrane filters and stored at −20 °C.

### 4.3. Enzyme Activity Assays

The enzyme activity assays were carried out using the supernatants collected on day 6, 8, 10, 12 and 14.

#### 4.3.1. Xylanase

Xylanase activity was measured using xylose as the reference standard. The reaction mixture contained 25 μL of 1% (*w/v*) suspension of xylan from oat spelts in 0.05 M sodium citrate buffer (pH 5.0) and 25 μL of crude enzyme sample. The reaction was carried out at 50 °C for 5 min. All reactions were carried out in triplicates. The amount of sugar released was measured using DNS method by adding 150 μL of DNS to stop the reaction and heating in a boiling water bath for 15 min [41]. The amount of sugar released was analyzed by measuring the absorbance at 540 nm using Synergy HTX Multi-Mode reader. The enzyme activity was expressed as micrograms of reduced sugar released per minute (U/min) under the given assay conditions.

#### 4.3.2. Protease

The protease activity was assayed using azocasein as the substrate [42,43]. The stable dye-labeled peptides and amino acids released into the reaction mixture as a result of enzymatic hydrolysis of azocasein were measured after the assay. Of the sample 25 µL was mixed with the same volume of azocasein (1 mg/mL) in 0.2 M Tris-hydroxymenthyl amino methane hydrochloride (Tris-HCL) buffer (pH 7.4). The resulting solution was incubated at 75 °C for 1 h. The reaction was terminated by adding 750 μL of 5% trichloroacetic acid (TCA) to the enzyme–substrate mixture. The coagulated protein was removed by centrifugation at 2000× *g* for 10 min at room temperature. The obtained supernatant was then added to a 0.5 N NaOH solution using a 1:1 (*v/v*) ratio and its absorbance was read at 440 nm using Synergy HTX Multi-Mode reader. The blank was obtained by mixing the TCA to the substrate prior to the enzyme addition. All reactions were carried out in triplicates. The activity of enzyme was expressed in arbitrary units where 1 unit of activity is equivalent to change the optical density of 0.01 nm per minute at 440 nm.

#### 4.3.3. Lipase

The lipase activity was measured using P-nitro phenyl palmitate (pNPP) as the substrate and p-nitrophenol as a standard reference [44]. The enzyme solution was added to the prewarmed phosphate buffer (0.05 M pH 8.0) containing 0.2% (*w/v*) sodium deoxycholate and 0.1% (*w/v*) gum Arabic to a final volume of 3 mL. The mixture was incubated for 5 min at 30 °C. After incubation, pNPP was added to reach a final concentration of 0.30 mM final concentration and the mixture was left to react for 3 min. All reactions were carried out in triplicates. The activity was measured at the wavelength of 405 nm using Synergy HTX Multi-Mode reader. The enzyme activity is expressed as the amount of p-nitrophenol produced per minute under the given assay conditions.

#### 4.3.4. Polygalacturonase

Polygalacturonase activity was assayed by measuring the reducing sugars using the dinitrosalicylic acid (DNS) method [41]. The reaction was carried out in 96 well flat bottom plates (Corning) with 50 µL of 0.1% polygalacturonic acid prepared in 0.05 M sodium acetate buffer (pH 5.0), 25 µL of buffer and 25 µL of culture filtrate. The reaction mix was incubated at 40 °C for 1 h in a water bath. All reactions were carried out in triplicates. The released reducing sugars were measured by the DNS method at wavelength of 440 nm using the Synergy HTX Multi-Mode reader and compared to the values using galacturonic acid standard curve. One unit of enzyme activity was expressed as micromoles of galacturonic acid produced per minute (U/min) under the given assay conditions.

#### 4.3.5. Pectin Lyase

Pectin lyase activity was measured with slight modifications to the previously reported protocol by Preiss and Ashwel [45]. The reaction mixture, which contained 33.5 µL of the crude enzyme sample, 33.5 µL of 0.5% citrus pectin, 66.6 µL of Tris HCl Buffer (0.05 M, pH 8.0) and 66.6 µL of calcium chloride, was incubated at 30 °C for 1 h in a 96 well flat-bottomed plates. The appearance of the red chromagen that has maximum absorbance at 548 nm was measured using the Synergy HTX Multi-Mode reader. All reactions were carried out in triplicates. One unit of activity was defined as the micromoles of galacturonic acid produced per min (U/min) under the given assay conditions.

#### 4.3.6. Chitinase

Colloidal chitin was prepared by the method of Wen et al [46]. One gram of chitin from shrimp (Sigma Aldrich) powder was added to 12 mL of HCl and kept overnight at 4 °C with continuous stirring. The mixture was added to 400 mL of ice-cold 95% ethanol with rapid stirring and was incubated at 25 °C overnight. The mixture was centrifuged at 5000× *g* for 20 min at 4 °C to collect the precipitate and was washed with sterile distilled water until it reached neutral pH. The prepared colloidal chitin was stored at 4 °C until the enzyme assay.

The chitinase activity was assayed by measuring the reducing ends of sugars. The assay was carried out by mixing 0.5 mL of both 1% colloidal chitin and the crude enzyme. After the incubation for 1 h, 3 mL of DNS were added to the mixture to terminate the reaction [41]. The mixture was boiled for 15 min and centrifuged to remove the insoluble chitin. Of the supernatant 200 µL were taken in the 96 well flat bottomed plate and the absorbance was measured at 540 nm using the Synergy HTX Multi-Mode reader. All reactions were carried out in triplicates. The reducing sugar was quantified from a standard curve using various concentrations of N-acetyl-D-glucosamine. 

#### 4.3.7. Endo beta-1, 4 glucanase (CMCase)

The endo-*β*-1,4-glucanase activity was determined by measuring the release of reducing sugar from carmellose (Sigma-Aldrich) with the 3,5-dinitrosalicylic acid (DNS) method [41]. The reaction mixture containing 10 μL of crude enzyme and 0.5% (*w/v*) carmellose in 30 μL of acetate buffer (0.05 M, pH 5.0), was incubated in a water bath at 60 °C for 10 min and the reaction was terminated by the addition of 10 μL of DNS. After 5 min of boiling, the reaction mixture was cooled down to room temperature in ice water and diluted using 160 μL of buffer. All reactions were carried out in triplicates. The absorbance was measured at 540 nm using the Synergy HTX Multi-Mode reader. One unit of endo-*β*-1,4-glucanase activity was expressed as micrograms of glucose released per minute (U/min) under the given assay conditions.

#### 4.3.8. Exo- β-1,4-glucanase (avicelase)

The reaction was carried out using 50 μL of 1% Avicel in 50 mM sodium citrate buffer (pH 5.0) and 50 μL of crude enzyme solution. After the incubation at 50 °C for 30 min, 200 μL of DNS was added to the mixture and boiled for 5 min [41]. The solution was cooled in ice for 5 min and the absorbance were measured at 540 nm using the Synergy HTX Multi-Mode reader. All reactions were carried out in triplicates. One unit of enzyme activity was expressed as millimoles of glucose released per minute (U/min) under the given assay conditions.

#### 4.3.9. β-. glucosidase

The assay was carried out using 4-nitrophenyl-*β*-D-glucopyranoside (PNPG) as the substrate [47]. The reaction mixture contained 50 μL of crude enzyme and 100 μL of 2 mM PNPG, incubated for 30 min at 50 °C. After the incubation, 100 μL of 0.1 M sodium carbonate was added to the reaction mix and the absorbance was measured at 405 nm using the Synergy HTX Multi-Mode reader. All reactions were carried out in triplicates. A standard curve was plotted with different concentrations of 4- nitrophenol. The enzyme activity was expressed as the amount of PNP produced in per minute (U/min) under the given assay conditions. 

#### 4.3.10. Cellulase (Filter Paper Assay)

Filter paper assay was carried out in flat-bottomed 96 well plates using the Whatman #1 filter paper of ¼-inch diameter circles cut out using a paper punch [48]. Of the diluted sample 80 μL in 50 mM citrate buffer was added to the substrate and incubated at 50 °C for one hour. After the incubation, 80 μL of DNS reagent was added and the mixture was boiled for 10 min, cooled in ice and the absorbance was measured at 546 nm. All reactions were carried out in triplicates. One unit of FPase was defined as the amount of enzyme that released 1 μmol of glucose per minute.

### 4.4. Quantification of Fumonisins, Beauvericin, Enniatins and Moniliformin in Rice and Liquid Cultures

Each fungal rice culture for the presence of mycotoxins was extracted and purified according to the method described by Urbaniak et al [37]. The liquid cultures from day 8 were subjected to mycotoxin quantification. The samples were collected from biological triplicates and were subjected to mycotoxin quantification.

Mycotoxin standards of high purity (fumonisins B_1_, B_2_, B_3_, enniatins A, A_1_, B, B_1_, beauvericin and moniliformin) were purchased from Sigma-Aldrich (Steinheim, Germany). Standard solutions of mycotoxins were prepared in methanol and kept in a freezer at −20 °C. LC/MS-grade organic solvents, water and other reagents were purchased from Sigma-Aldrich (Steinheim, Germany). Qualitative and quantitative analysis of mycotoxins were performed using a LC–PDA/TQD system consisting of an UPLC™ system (Acquity, Waters, Milford, MA, USA) coupled to a photodiode array detector (PDA) and a triple quadrupole mass spectrometer (TQD; Waters Micromass, Manchester, UK) according to the methods described in detail earlier [37,49]

### 4.5. Statistical Analysis

The results of β-glucosidase, pectate lyase and xylanase activities on 8th day of all fungal strains were subjected to statistical analysis along with the results of fumonisin quantification. The statistical analysis of the enzyme assay and fumonisin were carried out using Statistical Analysis Software (SAS). The null hypothesis significance testing was carried out using Welch’s T-test with *p* < 0.05. 

## Figures and Tables

**Table 1:** Statistical analysis of β-glucosidase, pectate lyase and xylanase activities on 8th day of Sokolik and Santana supplemented cultures of PEA1, PEA2, 34OX and 1757OX (results from triplicates).

Treatments			*p* Values	
		*β*-glucosidase	Pectate Lyase	xylanase
**PEA1**	Sokolik	0.007 *	0.012 *	1.09 × 10^−8^ *
	Santana	0.80	0.023 *	8.99 × 10^−7^ *
**PEA2**	Sokolik	0.0002 *	0.001 *	0.003 *
	Santana	0.899	0.024 *	2.99 × 10^−7^ *
**34OX**	Sokolik	1.91 × 10^−5^*	0.147	4.51 × 10^−5^ *
	Santana	0.0007 *	0.037 *	6.12 × 10^−5^ *
**1757OX**	Sokolik	0.0006 *	0.024 *	2.6 × 10^−5^ *
	Santana	0.001 *	0.034 *	0.83

* statistically significant.

**Table 2:** Amounts of FB_1_, FB_2_, FB_3_, beauvercin and moniliformin produced in rice cultures (mean values and standard error). Calculated from triplicate treatments.

	FB_1_ (µg/g)	FB_2_ (µg/g)	FB_3_ (µg/g)	Beauvericin (µg/g)	Moniliformin (µg/g)
PEA1	3593.93 ± 97.1	595.02 ± 23.4	72.10 ± 4.54	nd	nd
PEA2	2567.38 ± 164.9	362.75 ± 32.7	85.434.71	nd	nd
34OX	nd	nd	nd	29.12 ± 3.54	nd
1757OX	nd	nd	nd	52.40 ± 5.1	nd

**Table 3:** FB_1_ and FB_2_ produced in *F. oxysporum* liquid cultures upon addition of various substrates and pea extracts (mean values and standard errors). Calculated from triplicate treatments.

Treatments	FB_1_ (ng/µL)	FB_2_ (ng/µL)
34OX Control	0.2 ±. 0.007	0.012 ± 0.007
34OX + Glucose	0.036 ± 0.13	0.002 ± 0.002
34OX + Citrus pectin	0.019 ± 0.007	0.002 ± 0.002
34OX + Oat bran	0.004 ± 0.003	0
34OX + Sokolik extract	0.014 ± 0.005	0
34OX + Santana extract	0.005 ± 0.003	0
1757OX Control	0.004 ± 0.001	0.002 ± 0
1757OX + Glucose	0.003 ± 0	0.000 ± 0
1757OX + Citrus pectin	0.004 ± 0.001	0.000 ± 0
1757OX + Oat bran	0.000 ± 0	0
1757OX + Sokolik extract	0.001 ± 0.001	0
1757OX + Santana extract	0.001 ± 0.001	0

## Supplementary Materials

The following are available online at https://www.mdpi.com/2076-0817/9/11/976/s1. Supplementary information includes seven tables: Table S1–S7: Mean and Standard error (SE) for endo *β*-1, 4 glucanase (S1), exo *β*-1, 4 glucanase (S2), chitinase (S3), cellulase (S4), polygalacturonase (S5), protease (S6) and lipase (S7) activity assays of PEA1, PEA2, 34OX and 1757 OX upon addition of glucose, citrus pectin, oat bran, Sokolik and Santana extracts. Figure S1-S7: Graphical representations of endo *β*-1, 4 glucanase (S1), exo *β*-1, 4 glucanase (S2), chitinase (S3), cellulase (S4), polygalacturonase (S5), protease (S6) and lipase (S7) activity assays of PEA1, PEA2, 34OX and 1757 OX upon addition of glucose, citrus pectin, oat bran, Sokolik and Santana extracts. Table S8: Mean and Standard errors of FB_1_ and FB_2_ produced in *F. proliferatum.*
