# Supplementary material for: Induction of Fusarium lytic Enzymes by Extracts from Resistant and Susceptible Cultivars of Pea (Pisum sativum L.)
Source: Pathogens. 2020 Nov 23;9(11):976. doi: 10.3390/pathogens9110976 (PMC7700429; doi:10.3390/pathogens9110976)
Supplement: Supplementary file 1 [file pathogens-09-00976-s001.pdf]

Treatments		Day 2		Day 4		Day 6		Day 8		Day 10		Day 12		Day 14	
		Mean	SE	Mean	SE	Mean	SE	Mean	SE	Mean	SE	Mean	SE	Mean	SE
**PEA1**	Control	2.1	0.03	2.11	0.05	2.82	0.08	10.28	0.02	7.6	0	9.93	0.07	10.92	1.72
	Glucose					2.00	0.20	15.80	0.00	6.3	0.30	9.7	0.23	9.6	0.07
	Citrus pectin					1.63	0.03	7.17	0.03	6.4	0.20	12.1	0.03	11.77	0.10
	Oat bran					2.13	0.47	9.48	0.15	5.93	0	15.73	0.27	15.8	0.20
	Sokolik extract					2.50	0.17	8.57	0.03	7.7	0.03	11.83	0.57	10.4	1.47
	Santana extract					1.45	0.05	9.93	0.07	7.57	0.17	8.78	0.12	12.73	0.20
**PEA2**		Day 2		Day 4		Day 6		Day 8		Day 10		Day 12		Day 14	
	Control	1.98	0.18	1.57	0.03	1.27	0.20	7.23	0.03	9.50	0.17	9.90	0.10	11.43	1.50
	Glucose					2.05	0.12	7.57	0.10	10.40	0.40	12.12	0.15	23.62	0.02
	Citrus pectin					2.53	0.00	10.23	0.10	7.33	0.13	9.57	0.10	17.03	0.03
	Oat bran					2.57	0.37	8.23	0.10	5.87	0.07	11.53	0.00	9.97	0.03
	Sokolik extract					1.77	0.10	7.30	0.03	6.20	0.40	9.63	0.03	15.10	0.63
	Santana extract					2.17	0.23	8.17	0.43	7.70	0.30	14.77	0.43	18.47	0.07
**34OX**		Day 2		Day 4		Day 6		Day 8		Day 10		Day 12		Day 14	
	Control	3.20	0.07	1.90	0.23	2.53	0.20	7.17	0.23	6.30	0.30	13.17	0.17	11.03	0.03
	Glucose					1.87	0.40	8.63	0.03	5.35	0.15	14.80	0.33	11.57	0.10
	Citrus pectin					2.13	0.60	8.47	0.07	4.90	0.30	11.68	0.18	15.70	0.10
	Oat bran					1.87	0.13	8.50	0.03	5.42	0.65	11.90	0.03	18.40	0.07

	Sokolik extract					2.20	0.47	7.38	0.02	6.17	0.03	13.55	1.65	10.57	0.10
	Santana extract					1.80	0.47	7.05	0.05	6.33	0.20	14.60	0.47	12.18	0.05
		Day 2		Day 4		Day 6		Day 8		Day 10		Day 12		Day 14	
	Control	3.65	0.68	1.80	0.07	1.67	0.00	8.08	0.02	4.67	0.13	14.05	1.52	13.88	0.15
	Glucose					2.53	0.40	6.53	0.13	5.48	0.08	13.92	0.05	13.27	0.07
**1757OX**	Citrus pectin					2.12	0.05	8.10	0.63	5.20	0.13	11.97	0.10	12.43	0.23
	Oat bran					2.50	0.03	6.88	0.22	5.00	0.00	10.73	0.07	13.03	0.03
	Sokolik extract					2.57	0.03	7.22	0.05	5.63	0.03	12.47	0.07	12.60	0.00
	Santana extract					2.45	0.12	7.55	0.08	7.47	0.07	8.87	0.20	15.95	0.02

Table S1. Mean (micrograms of glucose released per minute (U/min)) and Standard error (SE) for endo *β*-1, 4 glucanase activity assay of PEA1, PEA2, 34OX and 1757 OX upon addition of glucose, citrus pectin, oat bran, sokolik and santana extract. The results were statistically insignificant (p>0.05).

Endo β-1, 4- glucanase

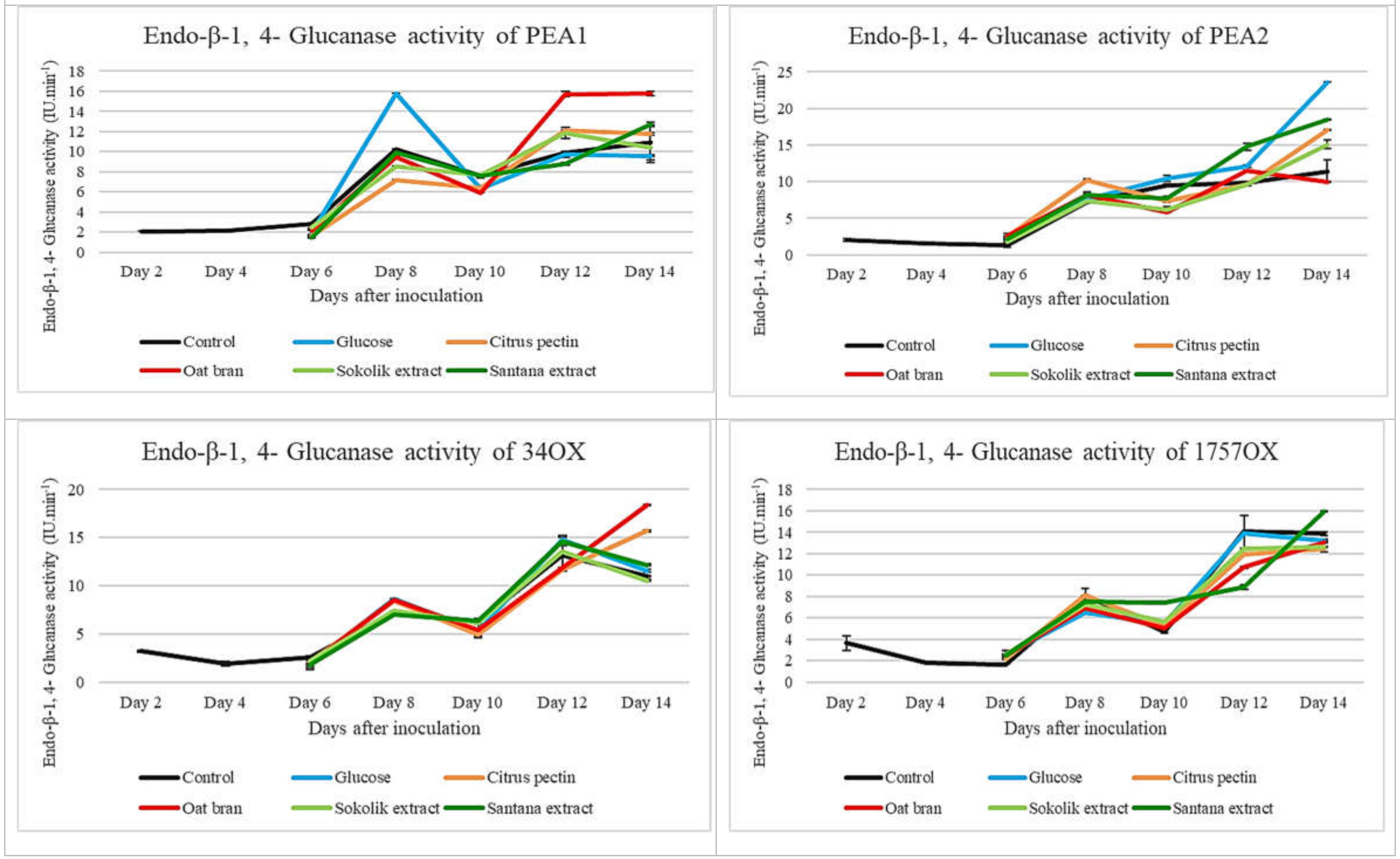

Figure S1. Endo β-1, 4 glucanase activity (micrograms of glucose released per minute (U/min)) of PEA1, PEA2, 34OX and 1757 OX upon addition of glucose, citrus pectin, oat bran, sokolik and santana extract. Error bar represent standard error.

	Treatments	Day 2		Day 4		Day 6		Day 8		Day 10		Day 12		Day 14	
		Mean	SE	Mean	SE	Mean	SE	Mean	SE	Mean	SE	Mean	SE	Mean	SE
**PEA1**	Control	0.15	0.01	0.22	0	0.13	0.05	0.13	0.01	0.08	0.03	0.12	0	0.07	0.01
	Glucose					0.08	0.01	0.13	0	0.09	0.01	0.12	0	0.12	0.04
	Citrus pectin					0.12	0.02	0.13	0.01	0.08	0.01	0.13	0.01	0.05	0.02
	Oat bran					0.16	0.03	0.09	0	0.08	0.01	0.10	0	0.10	0.02
	Sokolik extract					0.13	0.01	0.15	0.01	0.08	0.01	0.18	0.02	0.09	0.01
	Santana extract					0.10	0	0.16	0.02	0.11	0.01	0.17	0.00	0.13	0.03
**PEA2**		Day 2		Day 4		Day 6		Day 8		Day 10		Day 12		Day 14	
	Control	0.20	0.01	0.23	0.02	0.09	0.01	0.14	0.00	0.11	0.04	0.14	0.01	0.08	0.01
	Glucose					0.14	0.03	0.13	0.03	0.11	0.03	0.14	0.03	0.12	0.03
	Citrus pectin					0.13	0.03	0.15	0.04	0.09	0.01	0.10	0.02	0.13	0.04
	Oat bran					0.09	0.00	0.13	0.03	0.33	0.07	0.13	0.03	0.10	0.01
	Sokolik extract					0.13	0.01	0.23	0.00	0.14	0.01	0.15	0.04	0.08	0.01
	Santana extract					0.09	0.01	0.13	0.00	0.08	0.03	0.19	0.02	0.19	0.01
**34OX**		Day 2		Day 4		Day 6		Day 8		Day 10		Day 12		Day 14	
	Control	0.31	0.02	0.31	0.15	0.13	0.02	0.19	0.02	0.17	0.01	0.13	0.02	0.07	0.00
	Glucose					0.11	0.01	0.16	0.03	0.05	0.00	0.10	0.03	0.06	0.01
	Citrus pectin					0.11	0.01	0.21	0.01	0.07	0.02	0.11	0.03	0.06	0.01
	Oat bran					6.06	5.98	0.14	0.03	0.08	0.00	0.19	0.04	0.08	0.01

	Sokolik extract					0.11	0.02	0.12	0.01	0.10	0.02	0.10	0.00	0.10	0.00
	Santana extract					0.06	0.01	0.15	0.02	0.44	0.02	0.14	0.01	0.24	0.02
**1757OX**		Day 2		Day 4		Day 6		Day 8		Day 10		Day 12		Day 14	
	Control	0.14	0.00	0.22	0.04	0.09	0.01	0.08	0.01	0.10	0.00	0.11	0.02	0.10	0.02
	Glucose					0.07	0.00	0.04	0.02	0.09	0.02	0.03	0.03	0.06	0.01
	Citrus pectin					0.11	0.02	0.19	0.01	0.08	0.01	0.12	0.02	0.08	0.01
	Oat bran					0.06	0.00	0.23	0.02	0.04	0.01	0.07	0.01	0.09	0.01
	Sokolik extract					0.13	0.00	0.14	0.02	0.13	0.03	0.10	0.02	0.08	0.02
	Santana extract					0.19	0.00	0.14	0.01	0.22	0.07	0.17	0.04	0.13	0.02

Table S2. Mean(mM of glucose released per minute (U/min)) and Standard error (SE) for exo *β*-1, 4 glucanase (Avicelase) activity assay of PEA1, PEA2, 34OX and 1757 OX upon addition of glucose, citrus pectin, oat bran, sokolik and santana extract. Statistically significant values are given in red color (p<0.05).

Exo β-1, 4 glucanase (Avicelase)

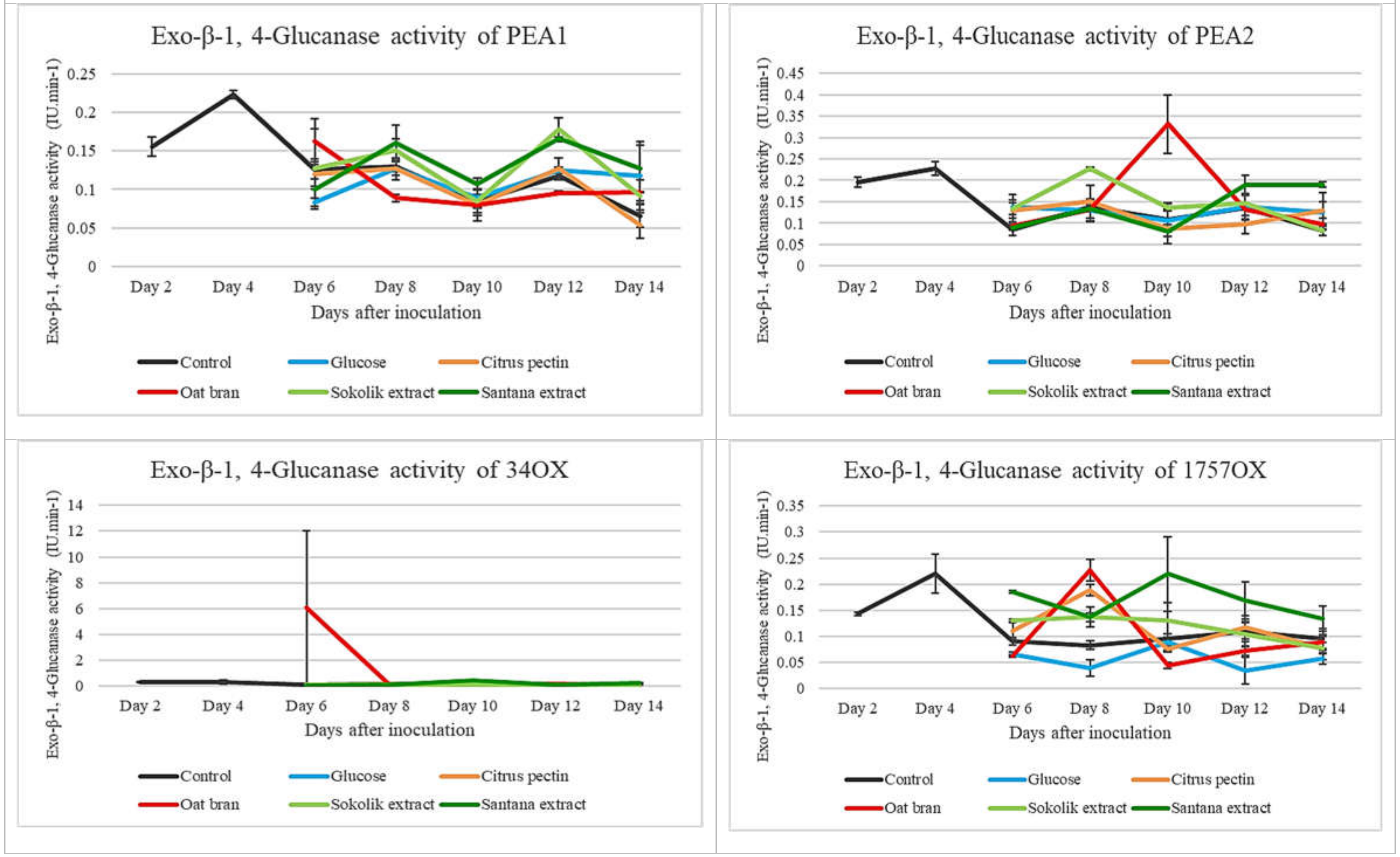

Figure S2. Exo β-1, 4 glucanase (Avicelase) activity (mM of glucose released per minute (U/min)) of PEA1, PEA2, 34OX and 1757 OX upon addition of glucose, citrus pectin, oat bran, sokolik and santana extract. Error bar represent standard error.

Treatments		Day 2		Day 4		Day 6		Day 8		Day 10		Day 12		Day 14	
		Mean	SE	Mean	SE	Mean	SE	Mean	SE	Mean	SE	Mean	SE	Mean	SE
**PEA1**	Control	0.09	0.001	0.014	0.001	0.009	0.0003	0.012	0.0003	0.014	0.0009	0.008	0.0009	0.009	0.0003
	Glucose					0.014	0.0023	0.011	0.0009	0.017	0.0060	0.009	0.0003	0.011	0.0007
	Citrus pectin					0.012	0.0015	0.011	0.0017	0.013	0.0037	0.009	0.0012	0.01	0.0009
	Oat bran					0.01	0.0013	0.009	0.0009	0.013	0.0021	0.008	0.0009	0.01	0.0012
	Sokolik extract					0.01	0.0007	0.01	0.0006	0.011	0.0000	0.009	0.0003	0.01	0.0006
	Santana extract					0.009	0.0003	0.016	0.0041	0.016	0.0035	0.009	0.0007	0.009	0.0006
**PEA2**		Day 2		Day 4		Day 6		Day 8		Day 10		Day 12		Day 14	
	Control	0.012	0.000883	0.011	0.000578	0.012	0.000334	0.011	0.001203	0.012	0.00252	0.008	0.000578	0.008	0.000334
	Glucose					0.011	0.001001	0.011	0.001203	0.017	0.001455	0.009	0.000334	0.01	0.000883
	Citrus pectin					0.01	0.000334	0.012	0.002407	0.012	0.000667	0.008	0.001001	0.01	0.001529
	Oat bran					0.01	0.000667	0.019	0.003218	0.012	0.000578	0.008	0.000334	0.011	0.001529
	Sokolik extract					0.01	0.000883	0.012	0.001001	0.009	0.000883	0.008	0.000578	0.011	0.001455
	Santana extract					0.011	0.001455	0.009	0.000334	0.01	0.000578	0.009	0.000667	0.011	0.001669
		Day 2		Day 4		Day 6		Day 8		Day 10		Day 12		Day 14	
	Control	0.019	0.004168	0.01	0.001203	0.009	0.000667	0.012	0.001203	0.014	0.002851	0.008	0.000334	0.011	0.001203
	Glucose					0.011	0.000883	0.012	0.001858	0.018	0.006514	0.008	0.000884	0.01	0.001156
	Citrus pectin					0.009	0.000334	0.013	0.002084	0.013	0.002312	0.01	0.001001	0.011	0.003004
	Oat bran					0.011	0.00203	0.011	0.001766	0.013	0.003059	0.009	0.000883	0.012	0.001734

**34OX**	Sokolik extract					0.01	0.001455	0.009	0.000883	0.009	0.001001	0.009	0.000334	0.008	0.000578
	Santana extract					0.01	0.001529	0.01	0.000883	0.009	0.000334	0.008	0.000667	0.012	0.000578
**1757OX**		Day 2		Day 4		Day 6		Day 8		Day 10		Day 12		Day 14	
	Control	0.01	0.000334	0.012	0.000578	0.01	0.001001	0.013	0.002336	0.011	0.000883	0.009	0.000883	0.009	0.001455
	Glucose					0.009	0.000883	0.01	0.000334	0.011	0.000334	0.01	0.000334	0.011	0.001203
	Citrus pectin					0.009	0.000883	0.011	0.000883	0.012	0.001001	0.01	0.000667	0.011	0.000334
	Oat bran					0.01	0.001156	0.01	0.001001	0.011	0.001529	0.008	0.000578	0.009	0.000334
	Sokolik extract					0.009	0.000884	0.008	0.000334	0.011	0.000334	0.01	0.000667	0.011	0.000883
	Santana extract					0.008	0.001001	0.009	0.000334	0.016	0.004848	0.01	0.001529	0.012	0.001766

Table S3. Mean(millimoles of glucose produced per minute (U/min)) and Standard error (SE) for chitinase activity assay of PEA1, PEA2, 34OX and 1757 OX upon addition of glucose, citrus pectin, oat bran, sokolik and santana extract. Statistically significant values are given in red color (*p*<0.05).

Chitinase

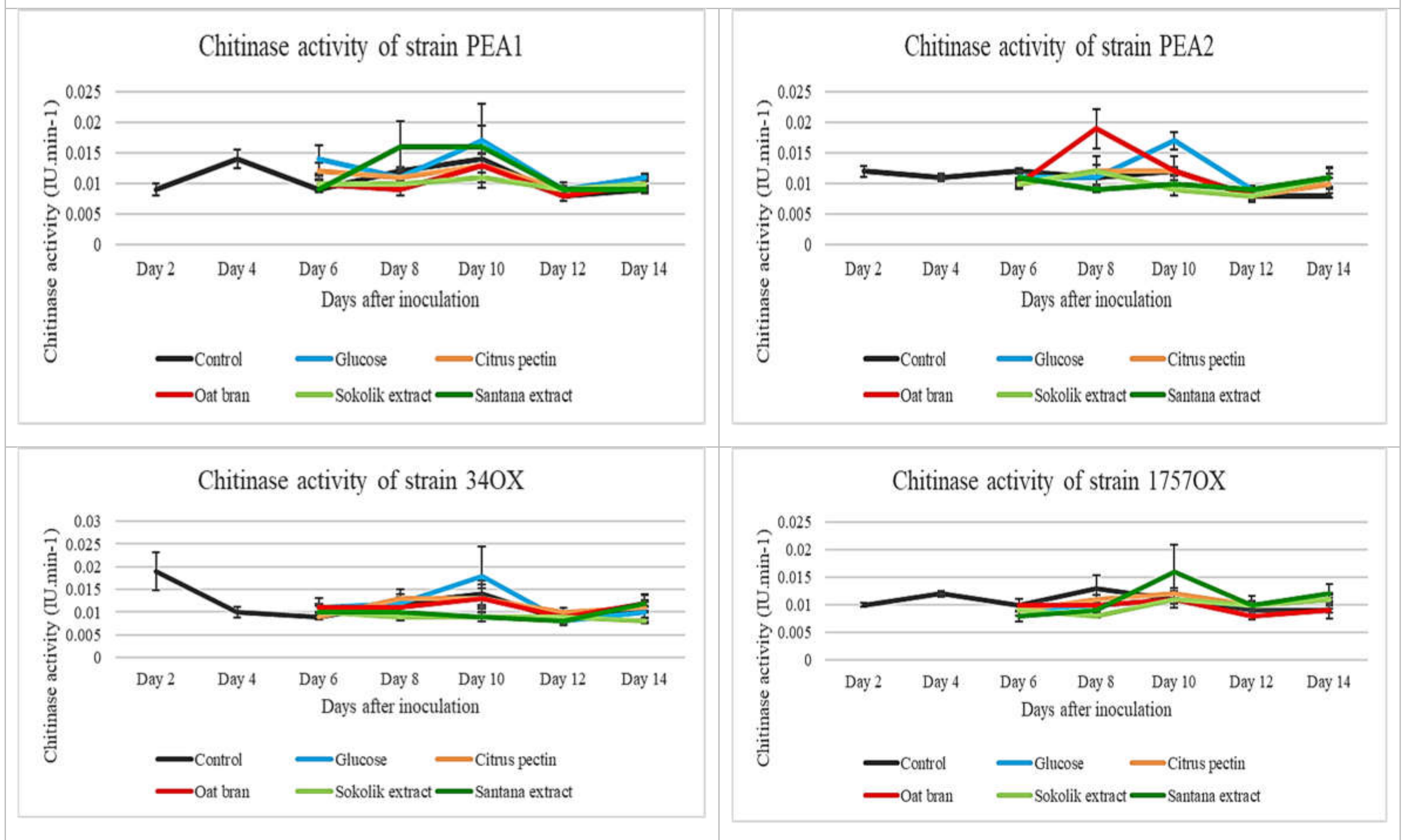

Figure S3. Chitinase activity (millimoles of glucose produced per minute (U/min)) of PEA1, PEA2, 34OX and 1757 OX upon addition of glucose, citrus pectin, oat bran, sokolik and santana extract. Error bar represents standard error.

	**Treatments**	**Day 2**		**Day 4**		**Day 6**		**Day 8**		**Day 10**		**Day 12**		**Day 14**	
		Mean	SE	Mean	SE	Mean	SE	Mean	SE	Mean	SE	Mean	SE	Mean	SE
**PEA1**	Control	27.51	1.02	29.41	1.17	25.68	1.84	42.87	1.83	35.41	1.68	25.69	1.04	33.51	2.50
	Glucose					25.87	2.72	36.27	0.67	35.41	1.68	25.89	1.21	32.85	0.57
	Citrus pectin					28.12	2.58	35.62	0.86	35.96	0.98	23.51	2.60	32.75	1.74
	Oat bran					27.72	1.16	36.33	1.21	38.44	0.39	24.73	3.34	26.39	3.40
	Sokolik extract					23.19	3.56	32.46	1.65	32.84	0.28	24.38	0.99	28.95	4.38
	Santana extract					24.48	3.94	34.72	2.66	31.83	1.17	23.48	2.08	27.55	2.10
**PEA2**		Day 2		Day 4		Day 6		Day 8		Day 10		Day 12		Day 14	
	Control	30.74	0.43	27.51	1.03	24.81	3.23	36.62	0.71	36.97	0.62	27.76	2.78	30.65	1.48
	Glucose					23.59	2.22	27.68	6.80	37.01	1.10	24.24	2.10	32.18	1.62
	Citrus pectin					30.60	1.32	30.35	4.35	37.77	1.38	71.62	1.01	32.09	0.48
	Oat bran					24.57	1.83	35.24	2.28	38.85	0.80	85.60	1.91	31.23	1.29
	Sokolik extract					24.03	3.52	32.93	1.22	36.05	1.61	30.35	1.36	29.64	2.71
	Santana extract					25.51	3.72	32.57	0.54	34.64	0.64	59.65	0.90	26.48	2.52
**34OX**		Day 2		Day 4		Day 6		Day 8		Day 10		Day 12		Day 14	
	Control	25.22	1.01	26.17	3.21	28.11	1.90	31.53	2.23	36.26	0.35	59.89	1.51	30.84	1.29
	Glucose					21.59	3.23	35.21	2.24	39.85	2.13	84.36	0.62	34.34	4.48
	Citrus pectin					26.61	1.27	31.02	4.17	40.44	2.96	53.63	1.59	32.85	0.57
	Oat bran					26.15	1.59	32.22	1.78	36.29	0.92	48.54	1.10	33.41	0.29

	Sokolik extract					26.87	1.67	30.54	0.90	36.26	0.35	23.13	0.14	33.96	2.06
	Santana extract					30.04	0.72	29.35	0.69	31.87	3.56	41.53	0.40	30.74	0.43
**1757OX**		Day 2		Day 4		Day 6		Day 8		Day 10		Day 12		Day 14	
	Control	33.38	1.97	28.92	0.77	22.91	2.47	32.59	0.74	35.29	0.87	38.63	0.28	34.78	1.71
	Glucose					33.13	0.50	33.01	1.71	35.62	0.86	70.66	0.41	34.72	1.35
	Citrus pectin					29.08	1.74	28.12	1.07	35.62	0.86	72.43	2.23	35.27	0.64
	Oat bran					28.80	4.21	31.32	2.37	40.53	1.24	23.04	1.15	37.31	0.37
	Sokolik extract					31.42	1.70	30.25	1.95	33.80	1.28	28.89	0.89	28.30	2.00
	Santana extract					29.11	2.45	24.70	2.32	30.79	2.78	26.66	1.99	31.55	2.20

Table S4. Mean(IU/min) and Standard error (SE) for cellulase (FPase) activity assay of PEA1, PEA2, 34OX and 1757 OX upon addition of glucose, citrus pectin, oat bran, sokolik and santana extract. Statistically significant values are given in red color ($p<0.05$).

Cellulase

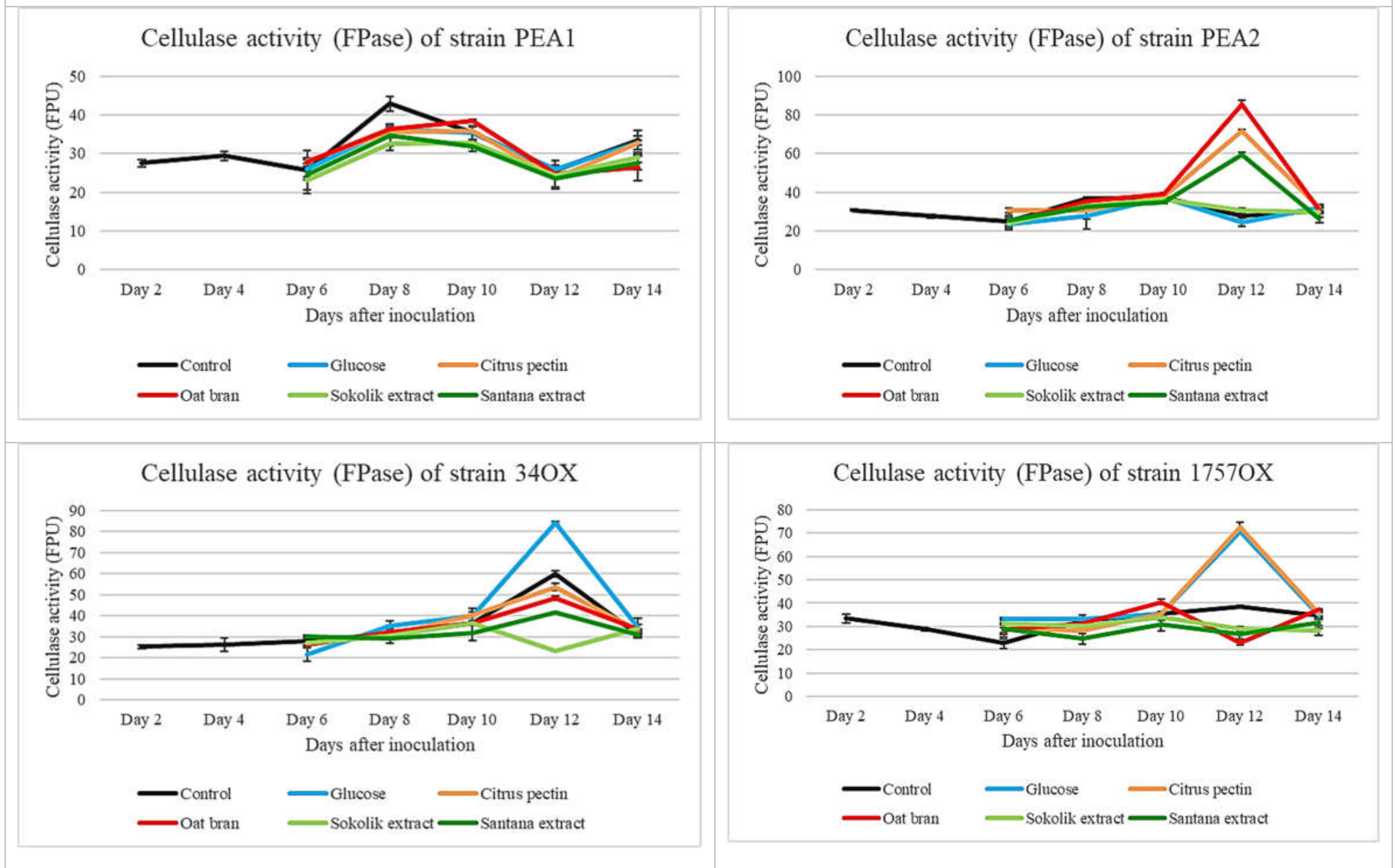

Figure S4. Cellulase (FPase) activity (FPU) of PEA1, PEA2, 34OX and 1757 OX upon addition of glucose, citrus pectin, oat bran, sokolik and santana. Error bar represents standard error.

	Treatments	Day 2		Day 4		Day 6		Day 8		Day 10		Day 12		Day 14	
		Mean	SE	Mean	SE	Mean	SE	Mean	SE	Mean	SE	Mean	SE	Mean	SE
**PEA1**	Control	5.17	0.09	5.07	0.16	5.26	0.05	4.38	0.26	4.65	0.06	4.86	0.11	5.06	0.25
	Glucose					5.27	0.29	4.80	0.04	4.44	0.05	5.45	0.02	5.17	0.11
	Citrus pectin					4.76	0.24	4.29	0.18	4.46	0.09	4.56	0.44	4.65	0.10
	Oat bran					4.70	0.07	4.87	0.16	4.66	0.17	5.05	0.11	4.91	0.32
	Sokolik extract					5.02	0.06	4.93	0.11	4.91	0.04	4.95	0.60	4.95	0.10
	Santana extract					5.14	0.34	4.74	0.14	4.70	0.16	4.22	0.15	4.88	0.11
**PEA2**		Day 2		Day 4		Day 6		Day 8		Day 10		Day 12		Day 14	
	Control	5.06	0.11	5.31	0.13	5.32	0.04	4.67	0.04	4.25	0.11	5.06	0.25	4.36	0.14
	Glucose					5.17	0.15	4.49	0.18	4.90	0.02	5.29	0.11	5.24	0.13
	Citrus pectin					5.11	0.09	5.07	0.08	4.74	0.44	5.36	0.10	4.80	0.37
	Oat bran					5.37	0.10	4.66	0.10	4.76	0.11	5.36	0.32	3.60	0.38
	Sokolik extract					5.61	0.26	5.06	0.06	4.54	0.60	5.57	0.10	5.56	0.09
	Santana extract					5.28	0.14	5.02	0.25	4.88	0.15	5.53	0.11	4.56	0.28
**34OX**		Day 2		Day 4		Day 6		Day 8		Day 10		Day 12		Day 14	
	Control	5.28	0.12	5.06	0.32	5.27	0.15	4.91	0.08	5.26	0.19	4.58	0.34	4.53	0.13
	Glucose					5.13	0.22	4.72	0.06	4.79	0.17	5.35	0.15	4.93	0.27
	Citrus pectin					5.22	0.19	4.35	0.25	4.95	0.06	5.46	0.23	4.94	0.04
	Oat bran					5.24	0.17	4.66	0.02	4.97	0.05	4.78	0.18	4.08	0.19

	Sokolik extract					5.08	0.10	5.06	0.17	5.07	0.25	5.10	0.05	4.75	0.38
	Santana extract					5.16	0.29	5.06	0.22	4.95	0.13	5.11	0.19	4.05	0.37
**1757OX**		Day 2		Day 4		Day 6		Day 8		Day 10		Day 12		Day 14	
	Control	5.35	0.17	4.16	0.18	5.36	0.61	4.69	0.17	5.30	0.23	4.98	0.13	4.96	0.19
	Glucose					5.08	0.07	5.93	0.08	5.19	0.43	4.95	0.22	4.91	0.14
	Citrus pectin					5.51	0.19	5.61	0.31	7.02	0.09	5.45	0.29	5.79	0.50
	Oat bran					5.43	0.47	6.75	0.31	5.22	0.19	5.26	0.15	5.34	0.51
	Sokolik extract					5.01	0.10	7.56	0.19	5.85	0.65	5.68	0.75	6.00	0.45
	Santana extract					5.03	0.13	4.98	0.04	4.90	0.24	5.43	0.84	5.46	0.53

Table S5. Mean(micromoles of galacturonic acid produced per minute (U/min)) and Standard error (SE) for polygalacturonase activity assay of PEA1, PEA2, 34OX and 1757 OX upon addition of glucose, citrus pectin, oat bran, sokolik and santana extract. Statistically significant values are given in red color (p<0.05).

Polygalacturonase

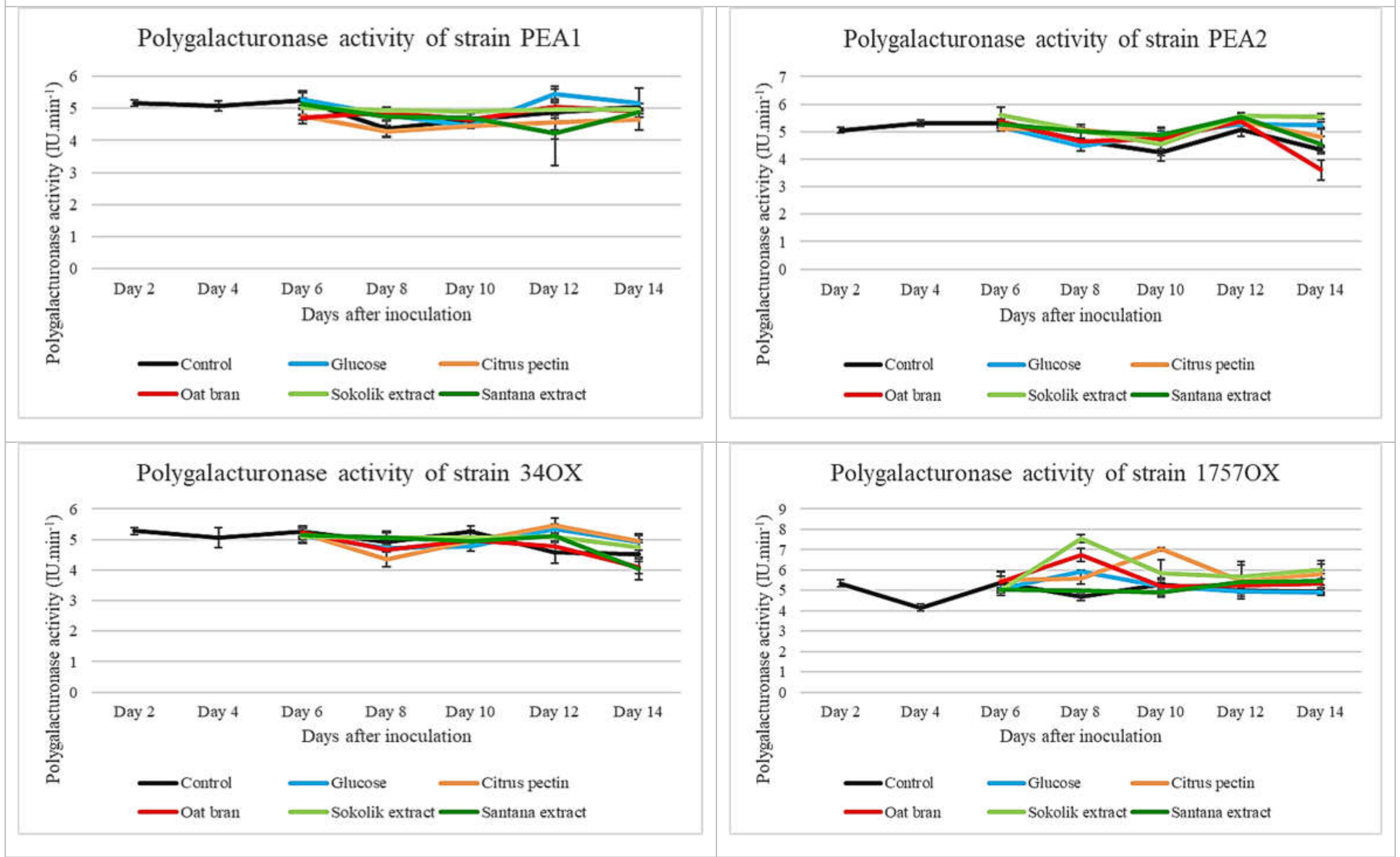

Figure S5. Polygalacturonase activity (micromoles of galacturonic acid produced per minute (U/min)) of PEA1, PEA2, 34OX and 1757 OX upon addition of glucose, citrus pectin, oat bran, sokolik and santana extract. Error bar represents standard error.

	**Treatments**	**Day 2**		**Day 4**		**Day 6**		**Day 8**		**Day 10**		**Day 12**		**Day 14**	
		Mean	SE	Mean	SE	Mean	SE	Mean	SE	Mean	SE	Mean	SE	Mean	SE
**PEA1**	Control	0	0	0	0	0	0	0.1	0.057	0.067	0	0.2	0.352	0	0.000
	Glucose					0.033	0.200	0.2	0.057	0	0.100	0.067	0	0	0.033
	Citrus pectin					0.6	0.057	0.033	0.033	0	0.033	0.1	0	0	0.033
	Oat bran					0.767	0.578	0.033	0.033	0.067	0	0.033	0.265	0	0.058
	Sokolik extract					1.167	0.537	0.033	0.033	0.2	0	0.067	0.200	0	0
	Santana extract					0.333	0.240	0.433	0.033	0	0	0.033	0.033	0	0
**PEA2**		Day 2		Day 4		Day 6		Day 8		Day 10		Day 12		Day 14	
	Control	0.033	0.033	0	0	1.3	1.251	0	0	0	0	0.033	0.033	0	0
	Glucose					0.167	0.088	0	0	0.167	0.166	0.233	0.185	0.033	0.033
	Citrus pectin					1.433	0.376	0.033	0.033	0.033	0.033	0.2	0.200	0.033	0.033
	Oat bran					0.2	0.152	0.167	0.088	0	0	0	0	0	0
	Sokolik extract					1.4	0.721	0.233	0.066	0.133	0.133	0.067	0.033	0.033	0.033
	Santana extract					0.267	0.266	0.067	0.033	0.067	0.066	0.033	0.033	0	0
**34OX**		Day 2		Day 4		Day 6		Day 8		Day 10		Day 12		Day 14	
	Control	0	0	0.467	0.006675	0.6	0.115	0.067	0.066	0	0	0.033	0.033	0	0
	Glucose					0.267	0.176	0.033	0.033	0.067	0.033	0.1	0.057	0.033	0.033
	Citrus pectin					0.2	0.200	0.067	0.066	0.033	0.033	0.033	0.033	0.067	0.033
	Oat bran					0.133	0.133	0	0	0	0	0.033	0.033	0	0

	Sokolik extract					0.933	0.376	0.433	0.23	0.033	0.033	0.067	0.033	0.133	0.033
	Santana extract					1.2	0.874	0.033	0.033	0.1	0.1	0	0	0.033	0.033
**1757OX**		Day 2		Day 4		Day 6		Day 8		Day 10		Day 12		Day 14	
	Control	0	0	0	0	0.367	0.185	0.033	0.033	0	0	0	0	0	0
	Glucose					0.033	0.033	0	0	0.167	0.08	0	0	0	0
	Citrus pectin					0.433	0.166	0	0	0.4	0.351	0	0	0	0
	Oat bran					0.8	0.251	0.1	0.1	0.033	0.033	0.067	0.033	0	0
	Sokolik extract					2.1	0.643	0.067	0.033	0.167	0.06	0.067	0.033	0.067	0.066
	Santana extract					0.033	0.033373	0.1	0	0.3	0.3	0.067	0.066	0.067	0.066

Table S6. Mean (IU/min) and Standard error (SE) for protease activity assay of PEA1, PEA2, 34OX and 1757 OX upon addition of glucose, citrus pectin, oat bran, sokolik and santana extract. The results are statistically insignificant ($p>0.05$).

Protease

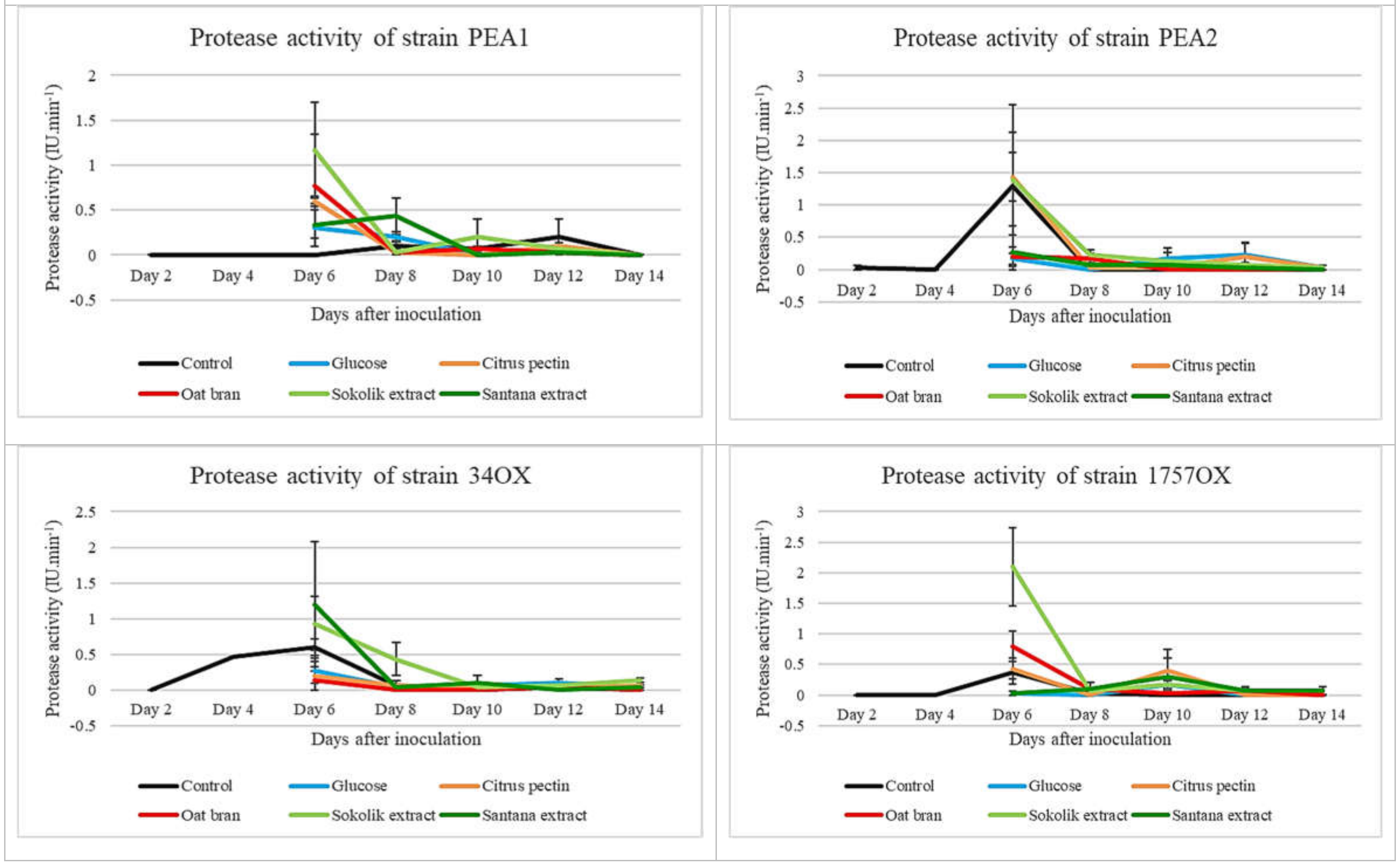

Figure S6. Protease activity (IU/min) of PEA1, PEA2, 34OX and 1757 OX upon addition of glucose, citrus pectin, oat bran, sokolik and santana extract. Error bar represents standard error.

	Treatments	Day 2		Day 4		Day 6		Day 8		Day 10		Day 12		Day 14	
		Mean	SE	Mean	SE	Mean	SE	Mean	SE	Mean	SE	Mean	SE	Mean	SE
**PEA1**	Control	0.16	0.05	0.21	0.00	0.38	0.06	0.55	0.06	0.87	0.16	0.70	0.18	1.55	0.08
	Glucose					1.16	0.20	0.74	0.08	0.89	0.11	1.00	0.10	0.77	0.14
	Citrus pectin					1.42	0.08	0.55	0.02	0.87	0.12	0.93	0.08	0.92	0.11
	Oat bran					1.35	0.09	0.52	0.05	1.12	0.20	1.10	0.05	0.97	0.16
	Sokolik extract					2.95	0.04	1.14	0.20	1.14	0.04	1.25	0.10	1.11	0.01
	Santana extract					0.60	0.02	1.17	0.08	1.60	0.09	1.64	0.02	0.83	0.05
**PEA2**		Day 2		Day 4		Day 6		Day 8		Day 10		Day 12		Day 14	
	Control	0.40	0.02	0.18	0.01	1.37	0.18	0.51	0.01	1.33	0.09	1.21	0.00	0.81	0.07
	Glucose					0.81	0.13	0.75	0.07	1.00	0.09	1.38	0.09	1.21	0.19
	Citrus pectin					0.98	0.11	0.41	0.14	1.48	0.08	1.08	0.23	1.08	0.11
	Oat bran					1.15	0.13	0.92	0.06	0.77	0.19	1.18	0.20	0.57	0.07
	Sokolik extract					1.46	0.10	1.20	0.13	0.92	0.21	1.32	0.19	0.93	0.12
	Santana extract					1.18	0.19	0.83	0.09	1.78	0.08	1.40	0.11	0.75	0.02
**34OX**		Day 2		Day 4		Day 6		Day 8		Day 10		Day 12		Day 14	
	Control	1.13	0.09	0.02	0.00	0.81	0.03	0.81	0.20	0.93	0.14	1.88	0.12	0.97	0.19
	Glucose					0.94	0.09	0.87	0.19	0.78	0.14	0.99	0.04	1.11	0.17
	Citrus pectin					0.85	0.01	1.09	0.18	0.68	0.10	1.22	0.09	0.96	0.08
	Oat bran					1.26	0.04	0.67	0.09	1.30	0.11	1.24	0.13	1.31	0.03

	Sokolik extract					2.71	0.15	0.97	0.17	0.79	0.21	1.12	0.02	0.59	0.15
	Santana extract					1.05	0.06	0.97	0.20	0.78	0.18	0.79	0.08	0.31	0.04
**1757OX**		Day 2		Day 4		Day 6		Day 8		Day 10		Day 12		Day 14	
	Control	0.20	0.02	1.29	0.04	0.99	0.14	0.54	0.05	0.90	0.01	0.85	0.07	0.73	0.10
	Glucose					1.16	0.16	0.51	0.02	1.13	0.09	0.61	0.12	0.76	0.12
	Citrus pectin					1.07	0.12	0.70	0.17	0.91	0.11	0.99	0.15	0.53	0.08
	Oat bran					0.95	0.02	0.59	0.11	0.82	0.10	0.99	0.13	0.64	0.05
	Sokolik extract					0.76	0.07	0.92	0.09	1.57	0.12	1.16	0.06	0.96	0.11
	Santana extract					0.95	0.23	0.75	0.10	1.71	0.03	0.70	0.16	1.22	0.06

Table S7. Mean(IU/min) and Standard error (SE) for lipase activity assay of PEA1, PEA2, 34OX and 1757 OX upon addition of glucose, citrus pectin, oat bran, sokolik and santana extract. Statistically significant values are given in red color ($p<0.05$).

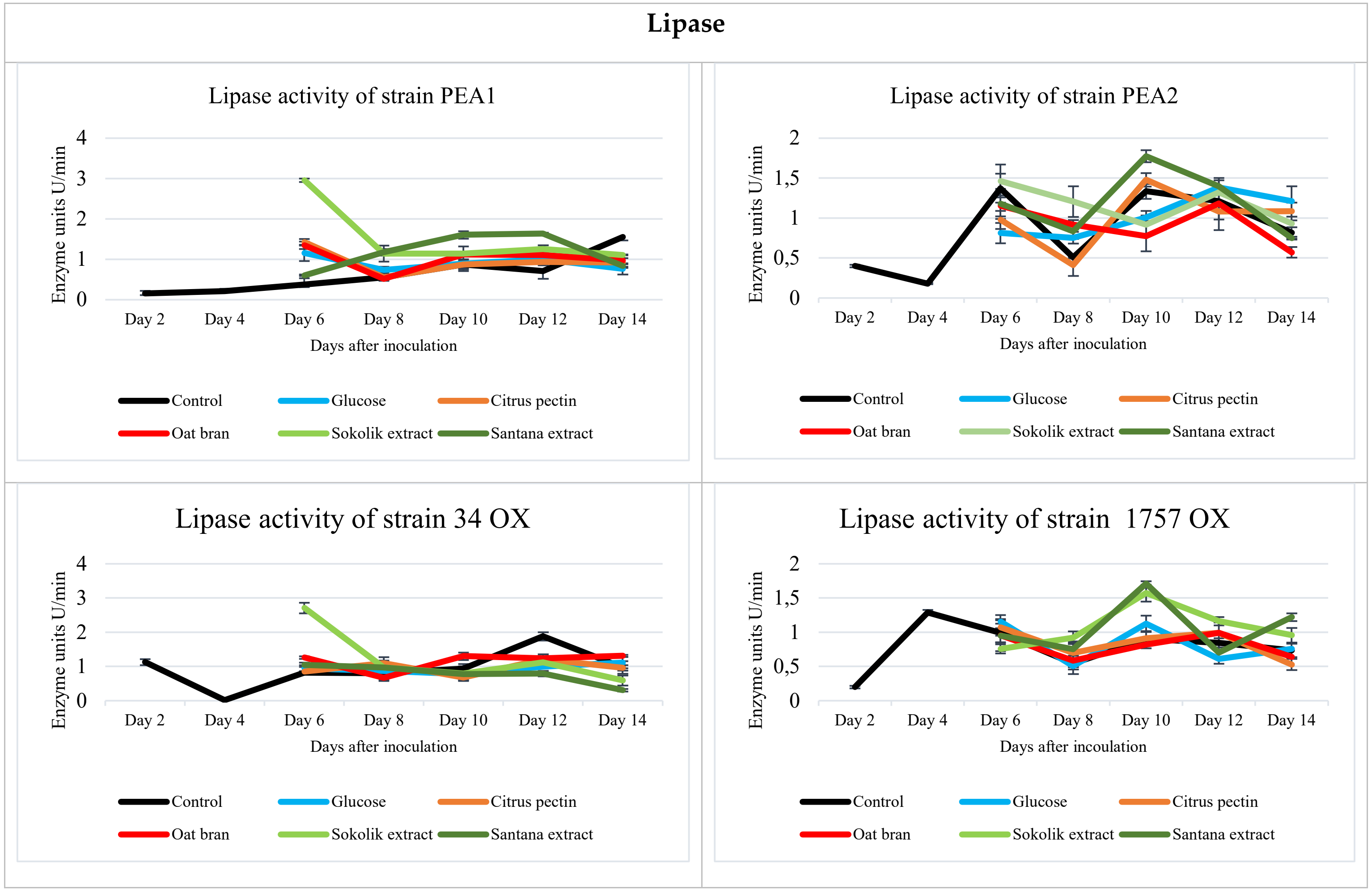

Figure S7. Lipase activity (IU/min) of PEA1, PEA2, 34OX and 1757 OX upon addition of glucose, citrus pectin, oat bran, sokolik and santana extract. Error bar represents standard error.

	FB$_1$												FB$_2$											
	Control		Glucose		Citrus pectin		Oat bran		Sokolik		Santana		Control		Glucose		Citrus pectin		Oat bran		Sokolik		Santana	
	**Mean**	**SE**	**Mean**	**SE**	**Mean**	**SE**	**Mean**	**SE**	**Mean**	**SE**	**Mean**	**SE**	**Mean**	**SE**	**Mean**	**SE**	**Mean**	**SE**	**Mean**	**SE**	**Mean**	**SE**	**Mean**	**SE**
**PEA1**	0.05	0.01	0.3	0.2	0.033	0.011	0.038	0.005	0.01	0.004	0.068	0.013	0.0126	0.001	0.018	0.001	0.003	0.001	0	0	0	0	0.003	0.001
**PEA2**	0.827	0.042	0.98	0.24	1.28	0.547	0.388	0.04	0.788	0.27	0.876	0.04	0.014	0.003	0.018	0.002	0.012	0.006	0.011	0	0.014	0.008	0.019	0.001

Table S8. FB$_1$ and FB$_2$ produced in *F. proliferatum* (PEA1 and PEA2) liquid cultures upon addition of various substrates and pea extracts (mean values and standard errors). Calculated from triplicate treatments. (The values are not statistically significant)
